# Conserved Sequence Preferences Contribute to Substrate Recognition by the Proteasome*[Fn FN2]

**DOI:** 10.1074/jbc.M116.727578

**Published:** 2016-05-17

**Authors:** Houqing Yu, Amit K. Singh Gautam, Shameika R. Wilmington, Dennis Wylie, Kirby Martinez-Fonts, Grace Kago, Marie Warburton, Sreenivas Chavali, Tomonao Inobe, Ilya J. Finkelstein, M. Madan Babu, Andreas Matouschek

**Affiliations:** From the ‡ Department of Molecular Biosciences and; the ¶ Center for Computational Biology and Bioinformatics, The University of Texas at Austin, Austin, Texas 78712,; the § Department of Molecular Biosciences, Northwestern University, Evanston, Illinois 60208,; the ‖ Medical Research Council, Laboratory of Molecular Biology, Cambridge CB2 0QH, United Kingdom, and; ** Frontier Research Core for Life Sciences, University of Toyama, 3190 Gofuku, Toyama-shi, Toyama 930-8555, Japan

**Keywords:** ATP-dependent protease, intrinsically disordered protein, proteasome, protein degradation, protein stability, protein targeting, protein turnover, ubiquitylation (ubiquitination), cellular protein abundance

## Abstract

The proteasome has pronounced preferences for the amino acid sequence of its substrates at the site where it initiates degradation. Here, we report that modulating these sequences can tune the steady-state abundance of proteins over 2 orders of magnitude in cells. This is the same dynamic range as seen for inducing ubiquitination through a classic N-end rule degron. The stability and abundance of His3 constructs dictated by the initiation site affect survival of yeast cells and show that variation in proteasomal initiation can affect fitness. The proteasome's sequence preferences are linked directly to the affinity of the initiation sites to their receptor on the proteasome and are conserved between *Saccharomyces cerevisiae*, *Schizosaccharomyces pombe*, and human cells. These findings establish that the sequence composition of unstructured initiation sites influences protein abundance *in vivo* in an evolutionarily conserved manner and can affect phenotype and fitness.

## Introduction

The proteasome controls the concentrations of thousands of regulatory proteins, removes misfolded and damaged proteins in cells, and digests foreign proteins to produce peptides that are displayed by the major histocompatibility complex (MHC) at the cell surface (1, 2). Proteins are targeted to the proteasome by ubiquitin chains, but these chains also have other biological functions (3, 4). The pattern through which ubiquitin moieties are linked and their number in the chains convey some targeting specificity. For example, chains of four or more ubiquitin moieties linked through Lys-48 of ubiquitin are thought to be the canonical proteasome targeting signal (5), whereas short tags of one ubiquitin or chains linked through Lys-63 are associated with membrane trafficking (6–8) and DNA repair (9).

Recent research shows that a much broader spectrum of ubiquitin linkages is associated with proteasome degradation (10, 11). In other cases, the same ubiquitin linkage can target proteins to different cellular processes (2, 3, 12–18). Even ubiquitin-tagged proteins that are recognized by the proteasome are not always degraded. It has been proposed that competition between different ubiquitin receptors (19) can protect some ubiquitinated proteins from proteasomal degradation. Additionally, disassembly of some polyubiquitin chains by specialized deubiquitinating enzymes on the proteasome can inhibit degradation (20).

Targeting information may also be encoded directly in the substrate protein itself. Efficient degradation requires the presence of an unstructured or disordered region in the substrate protein. The proteasome engages the substrate's disordered region to initiate unfolding and translocation to the proteolytic sites (21–24). The selection of the initiation site by the proteasome is part of the mechanism that confers specificity to degradation (25–28). Indeed, the absence of proteasomal initiation sites explains the unexpected stability of several natural proteins in yeast, such as the ubiquitin-conjugating enzyme Cdc34 and the proteasomal shuttle receptor Rad23 (27, 28). The requirement of initiation sites for degradation is also reflected in the half-lives of proteins measured in large scale proteomics experiments on yeast and mammalian cells (23, 28, 29). Proteins that contain predicted proteasome initiation regions have shorter half-lives than proteins that lack these regions (23, 28).

We recently investigated the proteasome's preferences for the amino acid sequence of its initiation sites *in vitro* by comparing the rates by which purified yeast proteasomes degraded a series of model proteins (28). However, it is possible that the proteasome's intrinsic preferences are overridden *in vivo*. For example, regulatory proteins such as p97/Cdc48/VCP may deliver already unfolded proteins to the proteasome (30–36). These considerations raise several questions. Are the initiation sequence preferences identified *in vitro* operational *in vivo*? If so, do the preferences affect protein abundance substantially and influence phenotype and cell fitness? How does the magnitude of the effect compare with that achieved by the regulation of ubiquitination? Are the sequence preferences for the initiation site conserved in different organisms?

Here, we investigate whether the proteasome has sequence preferences in cells using a scalable assay to monitor protein stability. We find that changes in the initiation sequence in model proteasome substrates tune protein degradation rates and adjust protein steady-state abundance over 2 orders of magnitude. These differences in abundance correspond to the dynamic range that is achieved by controlling ubiquitination. Modulating proteasomal initiation can change protein abundance sufficiently to affect cellular fitness by targeting His3 protein to degradation. We observe that the proteasome's sequence preferences are conserved between *Saccharomyces cerevisiae*, *Schizosaccharomyces pombe*, and cultured human cells (HEK293 cells). The sequence preferences reflect the binding affinity of the initiation regions to their recognition site on the proteasome and are related to specific physical and chemical properties of the amino acid sequences.

## Results

### 

#### 

##### Monitoring Proteasomal Degradation in Vivo

**FIGURE 1.**
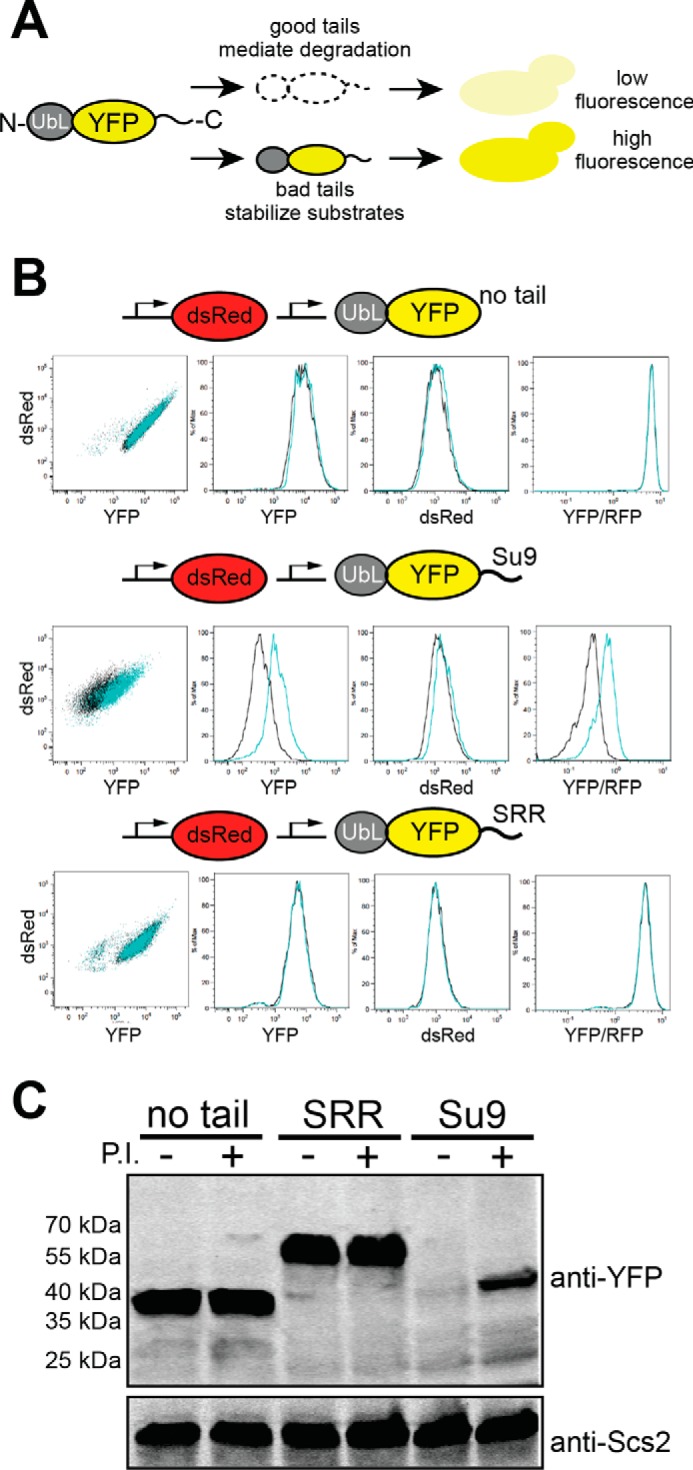
**Assessing proteasomal initiation in *S. cerevisiae*.** *A,* outline of the fluorescence-based degradation assay in *S. cerevisiae*. Proteasome substrates consisted of an N-terminal UbL domain derived from *S. cerevisiae* Rad23, followed by a YFP domain, and finally a disordered tail at the C terminus. Tails that allow the proteasome to initiate degradation resulted in a low cellular YFP signal, whereas tails that are not recognized by the proteasome led to a high YFP signal. *B,* cell fluorescence profiles of *S. cerevisiae* cultures expressing proteasome substrates with different initiation sequences monitored by flow cytometry. Cells were treated with proteasome inhibitor (100 μm bortezomib, *cyan* population) or DMSO (*black* population). 10,000 cells were collected in each flow cytometry run. *C,* cellular abundance of YFP substrates (UbL-YFP-tail) without an initiation sequence (no tail, Q) or with poor (SRR tail, E in supplemental Table S1) or effective (Su9 tail, P in supplemental Table S1) initiation sequences was assayed by Western blotting. The proteasome was inhibited with 100 μm bortezomib where indicated; the integral endoplasmic reticulum membrane protein Scs2, detected with a specific Scs2 antibody, served as the loading control.

To investigate protein targeting to the proteasome in *S. cerevisiae*, we assayed the abundance of fluorescent proteasome substrates by measuring total cell fluorescence. We constructed YFP variants with or without different degradation signals or degrons and expressed them from the constitutive *tpi1* promoter (37) on a CEN plasmid in *S. cerevisiae*. To correct for differences in plasmid copy number, transcription and translation levels, and cell size, we also expressed the RFP^3^ dsRed-Express2 (38) from a constitutive *pgk1* promoter (37) on the same plasmid (Fig. 1, *A* and *B*). The ratio of YFP over RFP fluorescence of individual cells served as a measure of the steady-state concentration of the YFP variants (39, 40). A high numerical value of the ratio of yellow fluorescence intensity to red fluorescence intensity (high YFP/RFP ratio) reports high YFP protein abundance and thus inefficient proteasomal degradation, and vice versa.

We targeted YFP to the proteasome by attaching the UbL domain of yeast Rad23 to its N terminus. The UbL domain is recognized by receptors on the proteasome (41–43), but the UbL domain and YFP lack disordered regions at which the proteasome can initiate degradation so that the UbL-YFP protein accumulated in cells and was easily detected by flow cytometry (Fig. 1*B*). Treating the cells with bortezomib, which partially inhibits the proteasome in *S. cerevisiae* (44, 45), did not increase UbL-YFP levels noticeably (Fig. 1*B*). Attaching a 51-residue C-terminal tail derived from subunit 9 of the *F_o_* component of the *Neurospora crassa* ATP synthase (Su9, sequence P in supplemental Table S1) to UbL-YFP reduced the yellow cell fluorescence to low levels slightly above the background fluorescence of cells not expressing YFP (Fig. 1*B*), suggesting that the UbL-YFP-Su9 protein was degraded efficiently. The red fluorescence of the RFP reference protein was not affected significantly (Fig. 1*B*). In the presence of bortezomib, UbL-YFP-Su9 fluorescence increased, showing that degradation depends on the proteasome, but to levels lower than UbL-YFP, as expected if proteasome inhibition is incomplete (Fig. 1*B*). Analysis of cell extracts by SDS-PAGE and Western blotting confirmed that the UbL-YFP-Su9 protein was depleted from cells in a proteasome-dependent manner and that the protein was degraded completely as no partially degraded protein fragments could be detected (Fig. 1*C*). Replacing the Su9 tail with a sequence consisting almost entirely of Ser residues (serine-rich region or SRR, sequence E in supplemental Table S1) stabilized the protein and restored UbL-YFP-SRR levels almost to those seen for UbL-YFP without an initiation region (Fig. 1*B*). Proteasome inhibition by bortezomib did not cause further increase in UbL-YFP-SRR levels (Fig. 1, *B* and *C*). These results show that only some disordered tails allow the proteasome to initiate degradation in cells.

##### Initiation Sequence Preferences in Vivo

**FIGURE 2.**
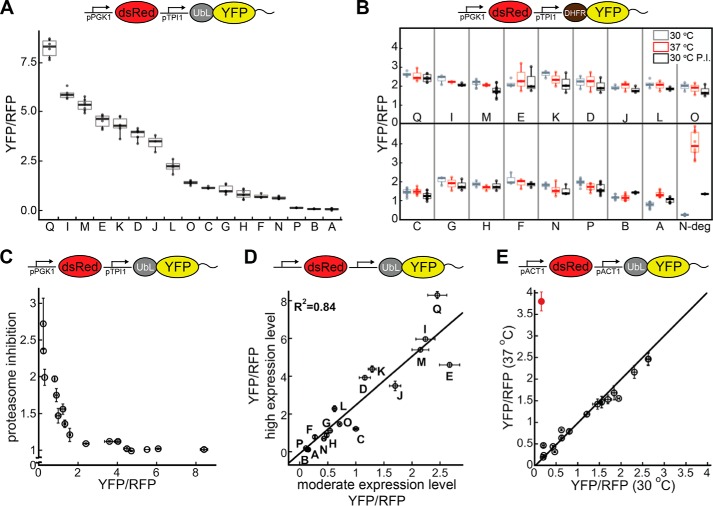
**Proteasomal preferences for initiation sequences in *S. cerevisiae*.** *A, boxplots* of corrected median cellular YFP fluorescence (median YFP/RFP values) for cultures expressing fluorescent proteasome substrates with different tails at their C termini (pPGK1 dsRed, pTPI1 UbL-YFP-tail; tail sequences shown in supplemental Table S1). *Whiskers* contain data within 1.5 interquartile range (IQR) of box. The IQR of the data is the difference between the 3rd quartile (75th percentile) and 1st quartile (25th percentile) and thus corresponds to the height of the box. *B, boxplots* (1.5 IQR whiskers) of YFP/RFP values for proteasome substrates in which the UbL domain was replaced with a DHFR domain (DHFR-YFP-tail) in the E1 temperature-sensitive strain *uba1-204* at the permissive (30 °C, *gray*) and restrictive (37 °C, *red*) temperatures and after proteasome inhibition (30 °C + 100 μm bortezomib, *black*). *N-deg*, YFP substrate with N-end rule degron. *C, graph plots* for the recovery of YFP/RFP values upon proteasome inhibition against the YFP/RFP values of each proteasome substrate. The recovery was calculated as the ratio of the median YFP/RFP values for cultures grown after addition of 100 μm bortezomib and DMSO. For stable YFP proteins, the YFP/RFP value does not change upon proteasome inhibition (recovery ≈1), and for well degraded proteins, the YFP/RFP value recovers as the proteasome is inhibited (recovery >1). *D, graph plots* median YFP/RFP values for cell cultures expressing proteasome substrates expressed at high levels (pPGK1 dsRed, pTPI1 UbL-YFP-tail) against the median YFP/RFP values for the same protein expressed at moderate levels (pACT1 dsRed, pACT1 UbL-YFP-tail). The correlation coefficient is calculated for a fit to a straight line. *E,* median YFP/RFP values of cell cultures expressing proteasome substrates with different tails at their C termini at moderate expression levels (pACT1 dsRed, pACT1 UbL-YFP-tail, *black circles*) in the E1 temperature-sensitive strain *uba1-204* at the permissive (30 °C) or the restrictive (37 °C) temperature. A ubiquitination-dependent N-end rule substrate is also shown (Ub-R-KK-YFP-Su9, *red circle*). The median YFP/RFP values for each construct were calculated from 10,000 cells collected in one flow cytometry run. Data points in *panels C, D,* and *E* represent mean values determined from at least three repeat experiments; *error bars* indicate S.E.

Next, we fused UbL-YFP to 14 additional disordered C-terminal tails (supplemental Table S1) and used the fluorescence assay described above to investigate intracellular degradation. The steady-state abundance of these proteins, as judged by YFP/RFP ratios, varied ∼70-fold between the most and least stable proteins (Fig. 2*A* and Table 1).

**TABLE 1:** **YFP/RFP ratios of constructs in *S. cerevisiae*, *S. pombe*, and mammalian cells (HEK293)** The median of YFP/RFP ratio for each construct was calculated from 10,000 cells collected in one flow cytometry run and used to indicate the abundance of the YFP substrate in yeast cells as described under “Experimental Procedures.” Data represent mean values and standard errors determined from at least three repeat experiments. ND is not determined; NS indicates derived from influenza A virus non-structural protein 1 (NS1); GRR indicates glycine-rich region (derived from human p105); NB indicates derived from influenza B virus glycoprotein NB; SNS indicates tandem repeat of SP2-NB-SP2; NBS indicates tandem repeat of NB-NB-SP2; DRR indicates aspartic acid (D)-rich region (derived from *S. cerevisiae* Cdc34); SP1 indicates peptide region 1 in influenza A virus M2 protein used to produce antisera; SP2 indicates peptide region 2 in influenza A virus M2 protein used to produce antisera; SPmix indicates tandem repeat of SP1 and SP2 (SP2-SP1-SP2-SP1-SP2); PEST indicates sequence from human IκBα; SRR indicates serine-rich region (derived from herpes virus 1 ICP4); Su9, derived from subunit 9 of *N. crassa* ATP synthase component *F_o_*; eRR, derived from *E. coli* lacI; ODC indicates derived from ornithine decarboxylase; 35 indicates derived from *S. cerevisiae* cytochrome *b*_2_.

Tail	Name	*S. cerevisiae*			*S. pombe*	HEK293
		pACT1 UbL-YFP-tail pACT1 dsRed	pTPI1 UbL-YFP-tail pPGK1 dsRed	pTPI1 UbL-GFP-tail pPGK1 dsRed		
A	35	0.15 ± 0.01	0.12 ± 0.02	0.13 ± 0.01	0.73 ± 0.06	0.20 ± 0.02
B	ODC	0.13 ± 0.01	0.13 ± 0.01	0.034 ± 0.004	1.0 ± 0.3	0.13 ± 0.02
C	Poly(G)	1.00 ± 0.05	1.22 ± 0.05	1.07 ± 0.08	ND	0.51 ± 0.01
D	GRR	1.2 ± 0.1	3.9 ± 0.1	1.9 ± 0.1	4.8 ± 0.2	0.83 ± 0.05
E	SRR	2.7 ± 0.2	4.6 ± 0.1	3.81 ± 0.09	6.54 ± 0.07	0.87 ± 0.02
F	NB	0.27 ± 0.01	0.79 ± 0.07	0.27 ± 0.02	2.8 ± 0.2	0.31 ± 0.02
G	NS	0.54 ± 0.03	1.12 ± 0.09	0.80 ± 0.03	2.3 ± 0.1	0.27 ± 0.02
H	SP1	0.48 ± 0.01	0.88 ± 0.08	0.63 ± 0.01	3.0 ± 0.1	0.47 ± 0.03
I	SP2	2.2 ± 0.2	6.0 ± 0.1	4.30 ± 0.06	5.65 ± 0.07	0.86 ± 0.04
J	SPmix	1.7 ± 0.1	3.5 ± 0.3	2.40 ± 0.07	5.94 ± 0.09	0.834 ± 0.004
K	SNS	1.29 ± 0.07	4.4 ± 0.2	2.7 ± 0.2	4.77 ± 0.07	0.70 ± 0.02
L	NBS	0.62 ± 0.02	2.3 ± 0.2	1.01 ± 0.07	5.2 ± 0.1	0.54 ± 0.02
M	DRR	2.2 ± 0.2	5.4 ± 0.1	4.6 ± 0.3	4.9 ± 0.2	0.76 ± 0.03
N	eRR	0.43 ± 0.02	0.70 ± 0.02	0.40 ± 0.01	1.10 ± 0.05	0.28 ± 0.02
O	PEST	0.71 ± 0.01	1.47 ± 0.04	0.98 ± 0.08	2.85 ± 0.08	0.21 ± 0.01
P	Su9	0.11 ± 0.03	0.19 ± 0.01	0.14 ± 0.02	1.7 ± 0.2	0.16 ± 0.01
Q	No tail	2.5 ± 0.2	8.3 ± 0.2	5.1 ± 0.3	6.8 ± 0.1	0.83 ± 0.06

Degradation of the YFP proteins depended on the UbL domain and was not due to ubiquitination of the disordered tails. *S. cerevisiae* encodes only one ubiquitin-activating enzyme (46), Uba1, and the temperature-sensitive *uba1-204* allele makes it possible to reduce protein ubiquitination substantially by shifting cells to the restrictive temperature (47). We replaced the UbL domain with a DHFR domain and fused the DHFR-YFP variants to the same 16 tails in a *uba1-204* strain. The steady-state levels of 14 of these proteins were similar both at the restrictive temperature and at the permissive temperature in the absence or presence of bortezomib (Fig. 2*C*). Shifting the *uba1-204* strain to the restrictive temperature does inhibit ubiquitin-dependent degradation of YFP substrate with a classic N-end rule degron (see below) more than 20-fold (Fig. 2*B*). These results indicate that most DHFR-YFP-tail variants are neither ubiquitinated nor degraded by the proteasome. The steady-state level of DHFR-YFP-35, which has a tail derived from the pre-sequence of *S. cerevisiae* cytochrome *b*_2_ (sequence A in supplemental Table S1), increased at the restrictive temperature and at the permissive temperature in the presence of bortezomib (Fig. 2*C*), suggesting that this sequence did become ubiquitinated to some extent. The steady-state level of DHFR-YFP-ODC, which has a tail derived from the 37 C-terminal amino acids of ornithine decarboxylase (sequence B in supplemental Table S1), increased by a small amount at the permissive temperature in the presence of bortezomib but was not ubiquitin-dependent (Fig. 2*B*). Proteasomal degradation of ornithine decarboxylase is known to be ubiquitin-independent (48, 49), suggesting that DHFR-YFP-ODC is directly targeted to the proteasome by the ODC tail. However, UbL-YFP-ODC was degraded substantially more efficiently than DHFR-YFP-ODC (Fig. 2, *A* and *B*), showing that tethering to the proteasome is important for robust degradation.

Inhibiting the proteasome with bortezomib increased accumulation of the least stable proteins by ∼3-fold, had no effect on the most stable proteins, and affected the proteins in-between proportionally to their abundance (Fig. 2*C*). Thus, degradation of unstable UbL-YFP-tail proteins was by the proteasome.

In principle, promoter strength might influence the proteasomal degradation of different proteins via aggregation or saturation of the folding or degradation machinery. To test this possibility, we expressed the set of UbL-YFP variants as well as RFP reference protein from two *act1* promoters (50) on the same CEN plasmid, which reduced cellular levels of a non-degraded UbL-YFP protein ∼5-fold compared with expression from the *tpi1* promoter (data not shown). At these lower expression levels, there was a 25-fold difference in abundance between proteins that degraded effectively (*e.g.* UbL-YFP-Su9; sequence P) and the proteins that degraded poorly (*e.g.* UbL-YFP-SRR; sequence E) (Table 1 and Fig. 2, *D* and *E*). Importantly, the steady-state levels of the UbL-YFP-tail proteins expressed from stronger and weaker promoters were highly correlated (*R*^2^ = 0.84; Fig. 2*D*). Thus, different expression levels did not cause a significant difference in the contribution of the tails to the initiation of proteasomal degradation. Again, degradation depended on the UbL domain and the proteasome (data not shown) and was not affected by ubiquitination, except for UbL-YFP-35 (sequence A in supplemental Table S1; Fig. 2*E*). UbL-YFP-35 was stabilized ∼2-fold when ubiquitination was inhibited, compared with a 25-fold stabilization when the tail was removed. Thus, the 35 tail was ubiquitinated to some extent, but its ubiquitination made a relatively small contribution to proteasome targeting.

##### Proteasome Initiation Tunes Degradation over a Similar Range as Ubiquitination

**FIGURE 3.**
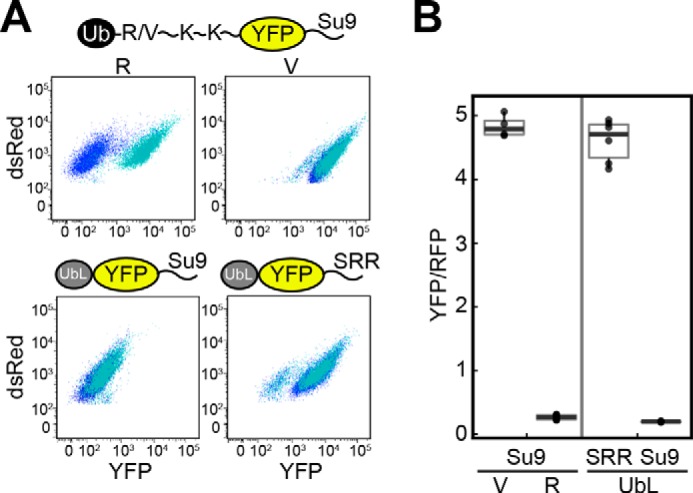
**Proteasome initiation tunes protein abundance over a similar range as ubiquitination.** *A,* cell fluorescence profiles of *S. cerevisiae* cultures expressing UbL-YFP-tail and N-end rule substrates. *Top row,* cells expressing N-end rule degron substrates with a destabilizing residue as the N degron (Ub-R-KK-YFP-Su9) or a stabilizing residue as the N degron (Ub-V-KK-YFP-Su9); *bottom row,* cells expressing UbL-YFP proteins with a tail that serves as an effective proteasome initiation site (Su9; P in supplemental Table S1) or a poor proteasome initiation site (SRR; E in supplemental Table S1). The proteins were expressed in the E1 temperature-sensitive strain *uba1-204* at the permissive temperature (30 °C, *blue*) or the restrictive temperature (37 °C, *cyan*). *B, boxplots* (1.5 IQR whiskers) of corrected YFP fluorescence (median YFP/RFP values) for cells expressing the N-end rule substrates and UbL substrates analyzed in *A*. The median YFP/RFP value for each construct was calculated from 10,000 cells collected in one run in flow cytometry.

Next, we asked whether initiation regions could modulate proteasomal degradation over the same dynamic range as achieved through the regulation of ubiquitination of a classic degron. We replaced the UbL domain of UbL-YPF-Su9 with an N-end rule degron consisting of a ubiquitin domain followed by a destabilizing (Arg) or stabilizing (Val) residue and a linker derived from *Escherichia coli lacI,* which contains two Lys residues (51, 52). In the cell, the ubiquitin domain is cleaved off by ubiquitin hydrolases, and an Arg residue leads to ubiquitination of the degron but a Val does not. Steady-state levels of the R-KK-YFP-Su9 protein were low and similar to those of UbL-YPF-Su9 (Fig. 3*A*, *blue* populations), whereas levels of the V-KK-YFP-Su9 protein were high and similar to those of UbL-YFP-SRR (Fig. 3*A*, *blue* populations). Inhibiting ubiquitination by shifting *uba1-204* cells to the restrictive temperature increased protein levels for R-KK-YFP-Su9 but did not affect V-KK-YFP-Su9, UbL-YFP-Su9 or UbL-YFP-SRR levels (Fig. 3*A*, *cyan* populations). Altering the N-end rule degron from Arg to Val changed YFP levels ∼26-fold (Fig. 3*B*), whereas modulating initiation by replacing the Su9 tail of UbL-YFP-Su9 with an SRR tail changed YFP fluorescence ∼24-fold (Fig. 3*B*). Thus, the identity of the initiation region can be as important as the regulation of ubiquitination in targeting proteins to proteasomal degradation.

##### Steady-state Levels Correlate with Degradation Rates

**FIGURE 4.**
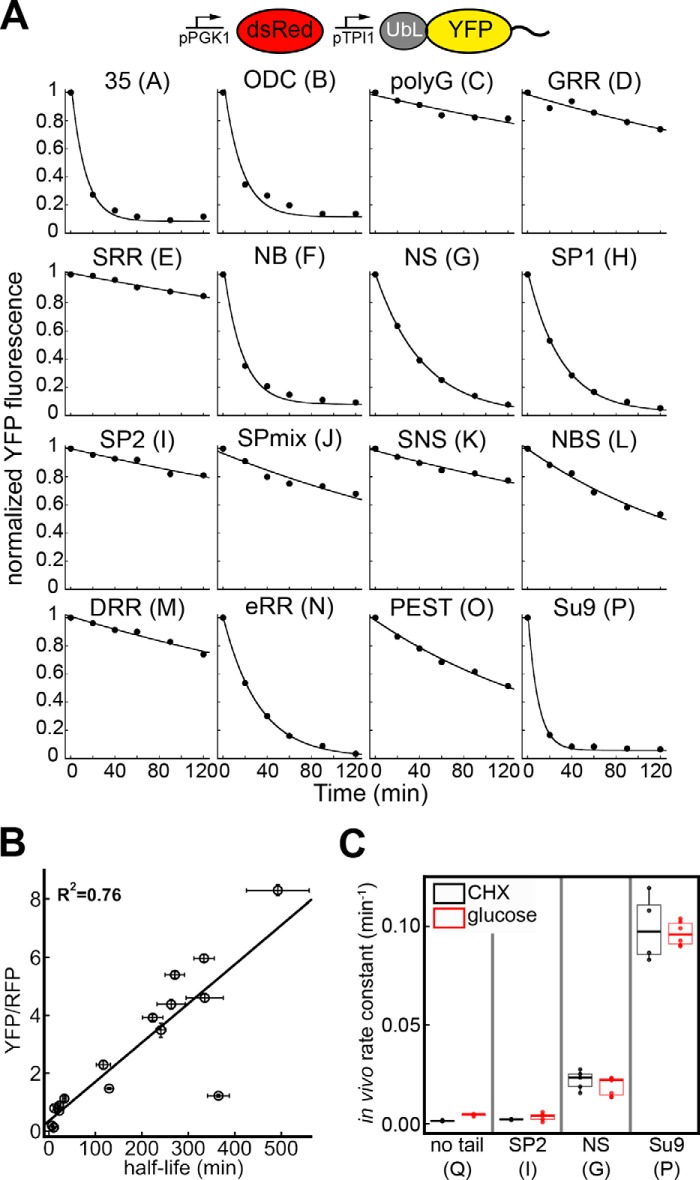
**Steady-state protein abundances correlate with degradation rates.** *A,* normalized time courses of YFP fluorescence illustrating *in vivo* degradation of UbL-YFP-tail constructs for 16 different tails after inhibition of protein synthesis by the addition of 125 μm cycloheximide. *Graphs* show one representative dataset of at least three independent experiments. *B,* relationship between protein abundance and half-life in yeast. The steady-state YFP/RFP ratios of fluorescent substrates with 16 different tails are plotted against the half-lives determined by nonlinear fitting of the data shown in *A* to a single exponential decay. The correlation coefficient is calculated for a fit to a straight line. Data points represent mean values determined from at least three repeat experiments; *error bars* indicate S.E. *C,* degradation rate constants of YFP substrates in yeast obtained from the cycloheximide chase experiment in *A* (*black*) and from a glucose shutdown assay (*red*) are shown. The median cellular YFP fluorescence for each construct at each time point was calculated from 10,000 cells collected in one run in flow cytometry; repeat experiments yielded the median values indicated in the *boxplots* shown (1.5 IQR whiskers).

The steady-state abundance of UbL-YFP-tail variants depended on the rates at which they were degraded by the proteasome in the cell. We measured degradation rates by inhibiting protein synthesis with cycloheximide and measured the amount of YFP substrate remaining over time (Fig. 4*A*). The half-lives of YFP substrates with different tails correlated well (*R*^2^ = 0.76) with their steady-state levels (Fig. 4*B*). The half-lives also varied over a similar dynamic range as the steady-state levels, with the least stable protein being degraded 68-fold faster than the most stable protein (Table 2). Thus, altering proteasomal initiation by changing the amino acid sequence of the disordered tails can tune degradation rates in the cell over a wide dynamic range. We conclude that the steady-state accumulation of the different proteins reflected the rates with which they were degraded by the proteasome.

**TABLE 2:** **Binding affinities of initiation sequences to the proteasome and *in vivo* degradation rate constants of fluorescent substrates (UbL-YFP-tail) in yeast** See Table 1 for definitions of names.

Name	*K_i_*	*In vivo* degradation rate constant
	μ*m*	*min*^−*1*^
35	36 ± 6	0.071 ± 0.004
ODC	28 ± 3	0.068 ± 0.007
Poly(G)		0.0019 ± 0.0001
GRR	130 ± 20	0.0033 ± 0.003
SRR		0.0022 ± 0.0003
NB	63 ± 12	0.07 ± 0.01
NS	158 ± 3	0.022 ± 0.002
SP1	86 ± 4	0.035 ± 0.004
SP2	322 ± 15	0.0021 ± 0.0001
SPmix		0.0029 ± 0.0001
SNS	133 ± 12	0.0028 ± 0.0003
NBS		0.0063 ± 0.0008
DRR		0.0026 ± 0.0002
eRR	36 ± 5	0.032 ± 0.001
PEST		0.0054 ± 0.0003
Su9		0.099 ± 0.008
No tail		0.0015 ± 0.0002

We also measured degradation rates for a subset of UbL-YFP-tail proteins by expressing them from a *gal1* promoter (37) and then shutting off expression by adding glucose. The rate constants determined in these experiments were very similar to the rate constants measured in the cycloheximide shut-off experiments for the same proteins expressed from the strong *tpi1* promoter (Fig. 4*C*).

##### Proteasomal Sequence Preferences Are Consistent for Different Proteins and Can Affect Fitness

Next, we tested whether altering proteasomal initiation of degradation could regulate the abundance of proteins other than YFP. We first replaced the YFP domain of UbL-YFP-tail proteins with jellyfish green fluorescent protein (GFP) (53), and found that the cellular levels of the UbL-GFP-tail and UbL-YFP-tail proteins were affected by the tail sequences in similar ways (*R*^2^ = 0.94 for a linear fit, Table 1).

**FIGURE 5.**
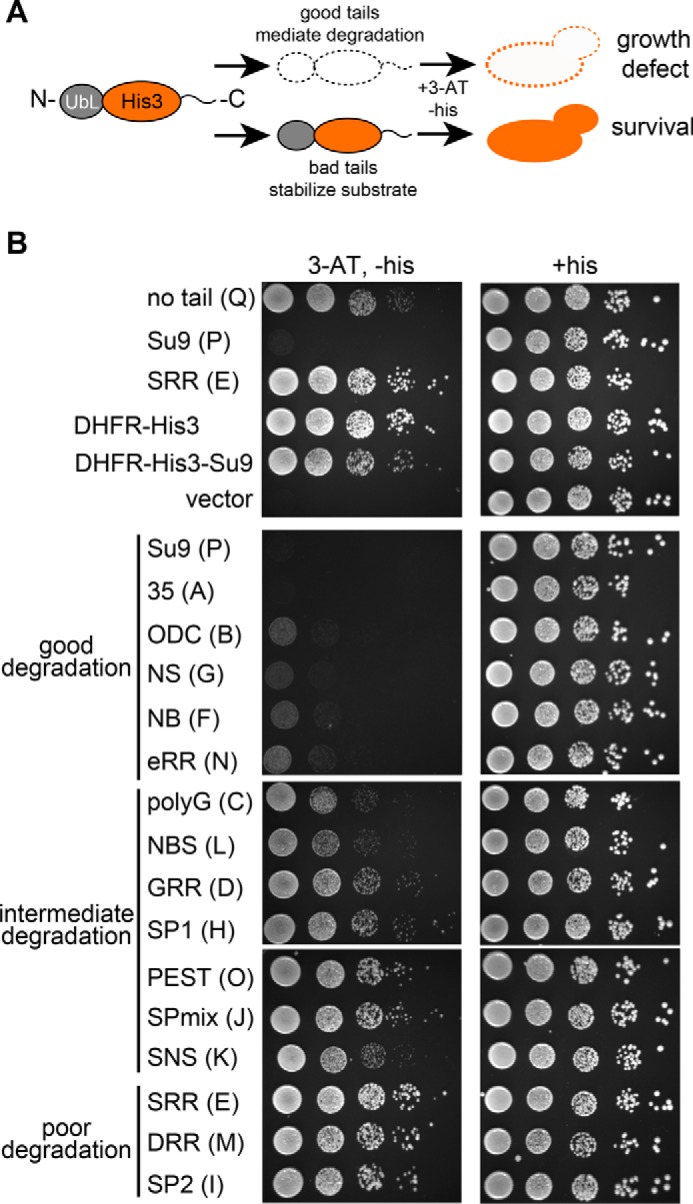
**Analysis of proteasome initiation using His3 protein degradation in yeast.** *A,* outline of the yeast growth assay. Imidazoleglycerol-phosphate dehydratase (His3) was targeted to the proteasome by fusing the UbL domain of *S. cerevisiae* Rad23 to its N terminus and different tails to its C terminus. Only tails that provide effective proteasome initiation regions mediate His3 protein degradation. In *his3* mutant cells, the absence of His3 protein causes growth defects in medium lacking histidine. *B,* cells expressing the indicated His3 fusion proteins in late log phase were serially diluted and stamped onto selective (+*3-AT*, −*his*) or non-selective (+*his*) synthetic medium. Plates were incubated at 30 °C for 3 days before imaging.

Fluorescent proteins do not occur naturally in *S. cerevisiae*, and their overexpression can have pleiotropic effects (50). To test a different protein, we chose *S. cerevisiae* His3, which is required for growth in medium lacking histidine (54). We constructed UbL-His3-tail proteins (Fig. 5) with the same 16 initiation regions analyzed in the YFP constructs, as well as a UbL-His3 protein without a tail and two His3 variants in which the UbL domain was replaced with a DHFR domain (DHFR-His3 and DHFR-His3-Su9). We tested whether expressing these proteins could complement growth of a *his3* mutant strain, using a competitive inhibitor of His3 (3-AT) to enhance assay sensitivity (55). Yeast with the parental control vector did not grow in the absence of histidine, but strains expressing the His3 variants that are not expected to be degraded, namely UbL-His3, DHFR-His3, and DHFR-His3-Su9, restored growth (Fig. 5*B*).

The growth phenotypes of the 16 different strains expressing UbL-His3-tail proteins fell into three broad groups, robust complementation, modest complementation, and poor or no complementation (Fig. 5*B*). The initiation sequences in the tails that prevented UbL-His3-tail proteins to complement in this assay also resulted in rapid degradation of the corresponding UbL-YFP-tail proteins. The tails that gave modest complementation resulted in intermediate UbL-YFP-tail degradation rates, and the tails that fully complemented resulted in slow degradation of UbL-YFP-tail proteins (*cf.* Figs. 2*A* and 4*A* with 5*B*). Expression of the His3 fusion proteins was not deleterious to yeast cells because supplementing the medium with histidine restored wild-type growth (Fig. 5*B*). In summary, the proteasome shows distinct preferences for the sequence of the disordered region in its substrate where it initiates degradation. These preferences are not dependent on the nature of the protein that is degraded. Furthermore, regulation of protein degradation by modulation of proteasome initiation can affect cell fitness.

##### Initiation Sequence Preferences Are Similar in Different Organisms

**FIGURE 6.**
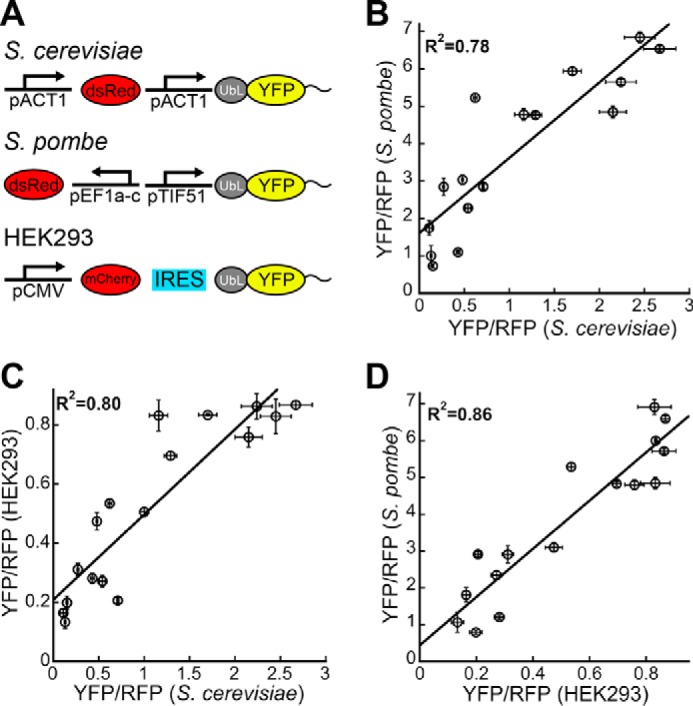
**Conserved sequence preferences in different species.** *A,* schematic representation of constructs used to assess proteasome targeting in *S. cerevisiae*, *S. pombe,* and cultured human cells (HEK293). In *S. cerevisiae*, YFP substrates and dsRed were both expressed from constitutive *act1* promoters on a CEN plasmid. In *S. pombe*, YFP substrates and dsRed were expressed from the constitutive promoters *tif51* and *ef1a–c*, respectively, after integration into genomic DNA. In HEK293 cells, mCherry and the YFP substrates were expressed from a single CMV promoter with their coding sequences separated by an internal ribosome entry site (*IRES*) on the mammalian expression vector pCDNA5. *B–D,* graphs plot corrected median cellular YFP fluorescence (median YFP/RFP values) for each construct expressed in two organisms against each other: *S. cerevisiae* and *S. pombe* (*B*), *S. cerevisiae* and HEK293 cells (*C*), and *S. pombe* and HEK293 cells (*D*). Correlation coefficients are calculated for fits to a straight line. The median YFP/RFP ratio for each construct was calculated from 10,000 cells collected in one flow cytometry run. Data points represent mean values determined from at least three repeat experiments; *error bars* indicate S.E.

The proteasome is evolutionarily conserved (56), although its processivity can vary substantially between different organisms (57). This raises the question whether the initiation rules for proteasomal degradation are the same for different organisms. To address this question, we tested the degradation of UbL-YFP-tail proteins in *S. pombe* and in cultured human HEK293 cells. To ensure proteasome targeting, we used the UbL domain of *S. pombe* Rhp23 (58) for the *S. pombe* experiments and the UbL domain of human HR23B (59) for the HEK293 experiments, as well as appropriate vectors, promoters, and red fluorescent proteins (Fig. 6*A*). The steady-state levels of the UbL-YFP-tail proteins, as assayed by YFP/RFP values, in the different organisms were highly correlated (Fig. 6, *B–D*). We conclude that the proteasomes of *S. cerevisiae*, *S. pombe*, and *Homo sapiens* share similar preferences for the amino acid sequence of their initiation sites.

##### Initiation Sequence Preferences Reflect Proteasome Affinity

**FIGURE 7.**
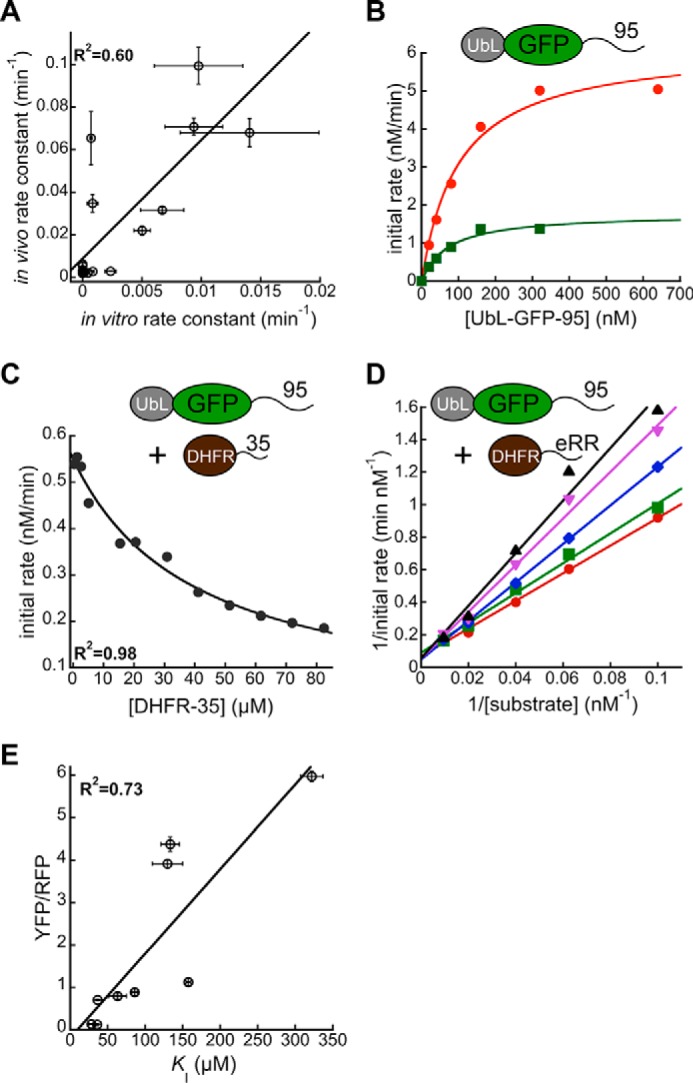
**Binding of initiation sequences to the proteasome.** *A,* correlation between substrate degradation rate constants *in vitro* and *in vivo*. Degradation rate constants *in vivo* for UbL-YFP-tail constructs as quoted in Table 2 are plotted against rate constants for degradation of Ub_4_-DHFR-tail substrates by purified yeast proteasome (adopted from Ref. 28). The correlation coefficient is calculated for a fit to a straight line. *B,* Michaelis-Menten plot for UbL-GFP-95 degradation by 10 nm (*green*) or 40 nm (*red*) purified *S. cerevisiae* proteasome. The substrate consisted of an N-terminal UbL domain derived from *S. cerevisiae* Rad23, followed by superfolder GFP and a 95-amino acid-long tail derived from *S. cerevisiae* cytochrome *b*_2_. *C,* initial degradation rates of UbL-GFP-95 in the presence of different concentrations of purified DHFR-35 by purified yeast proteasome are plotted and fitted to an equation describing competitive inhibition to calculate the inhibition constant *K_i_* for DHFR-35. *D,* initial degradation rates for different concentrations of UbL-GFP-95 in the presence of 0 μm (*red*), 2.6 μm (*green*), 10 μm (*blue*), 31 μm (*pink*), or 95 μm (*black*) DHFR-eRR. *E,* corrected median cellular YFP fluorescence (median YFP/RFP values) for cultures expressing fluorescent proteasome substrates with different tails at their C termini (pPGK1 dsRed, pTPI1 UbL-YFP-tail; Table 1) are plotted against the *K_i_* values for DHFR-tail constructs with the same tail (Table 2). The correlation coefficient is calculated for a fit to a straight line. UbL-GFP-95 degradation was followed by monitoring fluorescence intensity over time using a Tecan plate reader at room temperature as described under the “Experimental Procedures.” Tail sequences are shown in supplemental Table S1 (35, A; eRR, N).

Degradation rates of UbL-YFP-tail proteins in *S. cerevisiae* correlated with degradation rates measured *in vitro* for a set of proteins with the same tails as tested here (Fig. 7*A*). In the *in vitro* experiments, the tails were attached to a DHFR domain that was targeted to the proteasome by a tetra-ubiquitin chain, and these Ub_4_-DHFR-tail proteins were then degraded by purified *S. cerevisiae* proteasome (28).

To test whether initiation sequence preferences reflect a direct interaction with the proteasome, we developed an assay in which the different initiation regions compete with a substrate for degradation by purified *S. cerevisiae* proteasome. The substrate consisted of superfolder GFP with the UbL domain from *S. cerevisiae* Rad23 fused to its N terminus and a disordered region of 95 amino acids derived from *S. cerevisiae* cytochrome *b*_2_ fused to its C terminus. Degradation of this substrate by the proteasome in the presence of ATP, as assayed by loss of GFP fluorescence, followed Michaelis-Menten kinetics, with the *V*_max_ scaling linearly with proteasome concentration and the *K_m_* remaining constant (Fig. 7*B*). We fused the different disordered tails described above to the C terminus of *E. coli* DHFR and purified the proteins from *E. coli* by affinity chromatography. We first characterized DHFR-35 and DHFR-eRR (where eRR is derived from *E. coli* lacI) (see supplemental Table S1 for sequences; 35, A; eRR, N). Both tails supported robust degradation of UbL-YFP proteins in the cell (Figs. 2, 4–6). Increasing the concentrations of the DHFR-tail proteins progressively inhibited degradation of UbL-GFP-95 (Fig. 7, *C* and *D*). Inhibition was overcome by increasing the UbL-GFP-95 concentration, suggesting that UbL-GFP-95 and the DHFR-tail proteins compete for binding to the proteasome's receptor for the initiation region (Fig. 7*D*). The apparent inhibition constants (*K_i_*), with which the DHFR-tail proteins inhibited UbL-GFP-95 degradation, therefore reflected the affinity of the tails for the proteasome.

We then selected a subset of initiation regions and measured their ability to inhibit UbL-GFP-95 degradation (Table 2). The *K_i_* values ranged from ∼30 μm for the highest affinity interaction to ∼300 μm for the weakest interaction. The inhibition constants for different tails correlated well (*R*^2^ = 0.73) with their ability to support degradation, with tighter-binding tails leading to lower protein abundance in cells (Fig. 7*E*). Thus, the ability of disordered tails to initiate proteasomal degradation appears to be determined by their affinity for the proteasome.

##### Sequence Features of Initiation Regions

**FIGURE 8.**
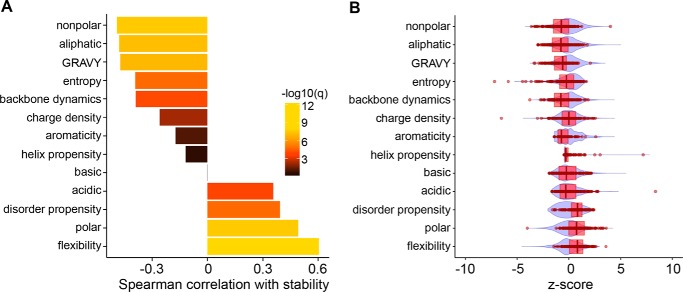
**Bioinformatics analysis of initiation sequences.** *A,* Spearman correlations between experimentally determined stabilities (measured by steady-state YFP/RFP ratios in Table 1 and supplemental Table S2 (pPGK1 dsRed, pTPI1 UbL-YFP-tail)) and physicochemical parameters of peptide sequences. The parameters for 115 sequences were calculated as described under the “Experimental Procedures.” Spearman correlation coefficients between the physicochemical parameters of the different sequences and YFP/RFP ratios were calculated. Statistical significance of these associations was accessed by pairwise *t* tests. The *p* values from *t* tests were then used to compute false discovery rate *q*-values by the Benjamini-Hochberg method (60), which are indicated in color (−log_10_(*q*)). *B,* comparison of the distribution of the parameters calculated for the 115 sequences analyzed in this study (box plots in *red*) and the same values calculated for the human proteome (violin plot in *blue*). A designed polypeptide library that covers the human proteome (T7-pep) (104) was used to represent the sequence characteristics of human proteome. The parameters were calculated as described under the “Experimental Procedures.”

Which sequence characteristics of disordered tails dictate the proteasome's initiation preferences? To study initiation preferences in greater detail, we used the 16 sequences characterized above (supplemental Table S1) as well as 99 additional sequences derived primarily from human and yeast proteins, and we tested their effects on steady-state levels of UbL-YFP-tail proteins *in vivo* (supplemental Table S2). We calculated a set of parameters of these sequences that report on their chemical or physical properties, such as hydrophobicity, charge, sequence complexity, flexibility, etc. (Fig. 8*A*). The sequence characteristics of the complete set of 115 sequences tested represent the properties of the human proteome well (Fig. 8*B*).

The scales for which the parameters reporting sequence properties are assessed were not developed with protein degradation in mind, and there is no *a priori* reason to assume a linear relationship between these parameters and degradation rates. Therefore, we took a nonparametric approach when relating the sequence parameters to protein degradation, using Spearman correlation coefficients to compare only the rank ordering of the different sequences by the various parameters with the rank ordering by YFP/RFP ratios. The correlations were tested for statistical significance (no correlation is the null hypothesis), with *p* values adjusted for false discovery by the Benjamini-Hochberg method (Fig. 8*A*) (60).

Consistent with our previous study (28), we found sequence complexity (measured as sequence entropy) to be correlated negatively with protein stability, suggesting that initiation sequences with biased amino acid compositions support proteasome initiation inefficiently (Fig. 8*A*). In addition to sequence complexity, hydrophobicity of the initiation region as measured by the fraction of aliphatic residues, the fraction of nonpolar residues, or the GRAVY algorithm (61) correlated negatively with stability (Fig. 8*A*). In contrast, sequence polarity (fraction of Asp, Glu, His, Lys, Asn, Arg, Ser, and Thr) and sequence acidity (fraction of Asp and Glu) correlated positively with stability (Fig. 8*A*). Thus, proteasome initiation seems more efficient at less hydrophilic and more hydrophobic regions.

Sequence dynamics also affect proteasome initiation and stiffer initiation regions appeared to promote better recognition and degradation. This relationship was found using two independent algorithms to predict sequence flexibility. The FLEXPLOT algorithm (62) predicts sequence flexibility by an algorithm calibrated using crystallographic B-factors, whereas the DynaMine tool predicts backbone dynamics through an algorithm calibrated by residue dynamics measured by nuclear magnetic resonance spectroscopy (63, 64). The two algorithms define their scales in opposite directions; the FLEXPLOT algorithm assigns numerically lower scores to stiffer sequences, and the DynaMine algorithm assigns numerically higher scores. Analysis based on the DynaMine algorithm resulted in a negative correlation between its score and stability, whereas the FLEXPLOT algorithm results in a positive correlation (Fig. 8*A*).

## Discussion

We find that the proteasome has distinct preferences for the amino acid sequence where it initiates degradation *in vivo*. Initiation regions tune protein abundance over 2 orders of magnitude, which is the same range as achieved by controlling ubiquitination of a classical N-end rule degron. Proteasomal initiation may regulate protein abundance over an even larger range, but our experimental system is limited by the level of protein expression and by signal over noise detection. Therefore, the proteasome's sequence preferences at the initiation step of degradation can contribute substantially to the regulation of protein abundance in the cell.

The ubiquitin code is ambiguous in that the proteasome can recognize polyubiquitin chains that typically target proteins to other processes. For example, polyubiquitin chains linked through Lys-63 are usually part of membrane trafficking processes, but they can target a protein to the proteasome both *in vitro* and *in vivo* (18). Proteins that are tagged with ubiquitin chains for fates other than degradation may have evolved to lack effective proteasome initiation sites to reduce the likelihood of degradation if they bind the proteasome. Conversely, if the proteasome is able to engage its target efficiently at the initiation region, even a weakly binding ubiquitin chain may induce degradation. Indeed, the presence of predicted proteasome initiation regions correlates with shorter protein half-lives *in vivo* (23, 27–29). Thus, natural variation in the unstructured regions of protein paralogs could tune protein abundance by modulating proteasomal initiation. Similarly, mechanisms that alter disordered regions such as alternative splicing (65, 66) could be used to tune protein abundance.

At the same time, the different polyubiquitin chains that target proteins to the proteasome physiologically are not necessarily synonymous. Cell cycle progression requires the degradation of regulatory proteins in the correct order. Some of these regulatory proteins are ubiquitinated by the same master ubiquitin ligase and ubiquitin-conjugating enzyme, but the polyubiquitin chains are synthesized with different efficiencies (67–69). The initiation step could contribute to substrate ordering if the late substrates, which are ubiquitinated less efficiently, also had less effective initiation regions and vice versa. Thus, the proteasome's initiation sequence preferences could contribute to the temporal control of degradation.

The proteasome's sequence preferences may also contribute to the accumulation of disease-related proteins. Several neurodegenerative diseases are associated with the buildup of proteins that appear to be targeted for degradation but escape destruction (70). Several of these proteins contain highly biased amino acid sequences. For example, the protein linked to Huntington disease (HTT exon1) is rich in proline and glutamine residues. It is in a disordered conformation but escapes proteasomal degradation even when ubiquitinated, apparently because of its biased sequence (28, 71, 72). Similar biased sequences also exist in the PRNP protein, which is associated with prion disease, in α-synuclein, which is associated with Parkinson disease and other disease-related proteins. The worst initiation regions analyzed here all share a biased amino acid composition with some amino acids strongly over-represented and others missing entirely (28). It is possible that the amino acid composition of the proteins associated with neurodegenerative diseases also makes them more difficult to be recognized by the proteasome and contributes to their accumulation in cells.

The proteasome's sequence preferences are conserved in *S. cerevisiae*, *S. pombe*, and cultured human cells, and they reflect a direct physical interaction between the initiation region in the substrate protein and the proteasome. The receptor of the initiation region on the proteasome is not known, but a likely site is the degradation channel that leads through the ATPase ring of the activator cap to the proteolytic chamber in the core of the proteasome.

The degradation channel is lined by two sets of loops, the P1 and P2 loops, and their sequences are conserved between eukaryotic proteasomes and bacterial AAA^+^ proteases, which fulfill similar functions as the proteasome (2, 73–79). The biochemical mechanism of the *E. coli* AAA^+^ protease ClpXP is particularly well understood (79). The P1 loops undergo conformational changes during ATP-dependent proteolysis and are thought to act as paddles that move the polypeptide chain through the pore (77, 78, 80–82) toward a second binding site formed by the P2 loops (80). Binding to the second site may prevent backsliding of the polypeptide chains between strokes of the P1 paddles (80).

By analyzing ∼100 different initiation regions, we were able to determine some sequence properties that govern how well the proteasome is able to initiate degradation. For example, stiffer initiation regions lead to more rapid degradation, perhaps because they increase the distance through space that an initiation region can explore. The P1 loops are located 1–3 nm from the entrance to the degradation channel as judged by the proteasome structure (83–86) so that stiffer sequences may be able to reach the P1 loops and engage the translocation motor more efficiently than more flexible sequences. The preference for hydrophobic initiation sequences may reflect their ability to interact with the P1 paddles, which have the consensus sequence aromatic-hydrophobic-Gly. The stabilizing effect of acidic sequences may be a reflection of weaker interaction between these sequences and the P2 loop region, which contains Glu and Asp in the proteasome. The sequence preferences could also be a reflection of consensus sequences in initiation regions that are recognized by their receptor on the proteasome. The relationship between sequence complexity and proteasome degradation could then be explained by the fact that biased sequences would be less likely to contain sequences that resemble the consensus motif than diverse sequences (28).

Bacterial AAA^+^ proteases can recognize their substrates and initiate degradation at the same sequences. These targeting sequences fulfill both the recognition and initiation functions, although recognition can be enhanced by targeting adaptors (87). Some 20 ClpXP degrons have been described in *E. coli* (88). The degrons are 10–12 amino acids long (88) and thus appear to be shorter than the proteasome initiation regions discussed here. The shorter length is consistent with the fact that the P1 loops in ClpX are also closer to the entrance to the degradation channel than in the proteasome. One of the bacterial targeting sequences, the SsrA tag, is particularly well characterized and binds ClpXP with ∼1 μm affinity (79). The sequence of the SsrA degron is precisely defined in the sense that single amino acid substitutions can reduce degradation drastically (88). Some eukaryotic proteins are targeted to the proteasome in the absence of ubiquitin, with ODC as the best characterized example (89). However, even the ODC initiation region binds the proteasome with considerably lower affinity than the SsrA tag. Efficient ODC degradation requires the protein Antizyme to serve as an adaptor that increases ODC's affinity to the proteasome (90, 91) in analogy to the bacterial adaptor proteins. So far the proteasome initiation regions seem to be less precisely defined than the ClpXP degrons (88), and the results summarized here did not reveal specific consensus motifs that are recognized by the proteasome. Larger sequence libraries will have to be analyzed to reveal such consensus sequences, if they exist.

In summary, we show that *in vivo* the proteasome has pronounced preferences for the amino acid sequence of its targets at the site where it initiates degradation. The selection of initiation sites represents a degradation code that is embedded within the target proteins. This initiation code is as important and operates in parallel to the ubiquitin code. Its evolutionary conservation suggests that the mechanism may alter the abundance distribution of the proteome in a wide variety of cells and organisms, affecting diverse genetic and regulatory processes.

## Experimental Procedures

### 

#### 

##### Substrate Proteins

Proteasome substrate proteins were derived from *E. coli* DHFR, *S. cerevisiae* His3, superfolder GFP (53), and a rapidly maturing derivative of YFP (a gift from B. S. Glick (University of Chicago)). N-terminal UbL domains from *S. cerevisiae* Rad23 or its homologs in *S. pombe* (Rhp23) and human cells (human HR23B) were connected to His3, GFP, or YFP by the linker sequence (VDGGSGGGS). C-terminal tails were attached through a 2-amino acid linker (Pro-Arg), and the amino acid sequences of the tails are shown in supplemental Tables S1 and S2.

##### Protein Expression and Purification

Yeast proteasome was purified from *S. cerevisiae* strain YYS40 by immunoaffinity chromatography using FLAG antibodies (M2-agarose affinity beads, Sigma), as described previously (92). Proteasome preparations were analyzed by SDS-PAGE and compared with published compositions (83). Each proteasome preparation was tested for activity by measuring degradation of the proteasome substrate UbL-DHFR-95 and for contamination by proteases by testing for stability of proteins that lack a proteasome-binding tag (DHFR-95) as described previously (57, 93).

The substrate (UbL-GFP-95) and competitor proteins (His_10_-DHFR-tail) used in *in vitro* inhibition assays were overexpressed in *E. coli* and purified using standard methods. Constructs were cloned into a pET3a vector and expressed from the T7 promoter in *E. coli* strains BL21(DE3)pLysS or Rosetta(DE3)pLysS (Novagen). Proteins were purified by TALON metal affinity beads (catalog no. 635502, Clontech) following the manufacturer's instructions. Purified proteins were dialyzed into buffer containing 50 mm Tris, pH 7.4, 300 mm NaCl, and 1% glycerol for storage. Protein concentrations were determined by measuring light absorbance at 280 nm and using extinction coefficients predicted from the proteins' sequence (ExPASy's ProtParam). The integrity and purity of proteins were evaluated by SDS-PAGE.

##### Competition Assays

The degradation of a fluorescent substrate protein (UbL-GFP-95) *in vitro* was monitored by measuring GFP fluorescence intensity over time in 384-well plates using a plate reader (Infinite M1000 PRO, Tecan) as described previously (94). Assays were carried out at 30 °C by adding fluorescent substrates at the indicated concentrations to 40 nm purified yeast proteasome in a reaction buffer (50 mm Tris, 5 mm MgCl_2_, 2.5% glycerol, 1 mm ATP, 4 mm DTT, 0.2 mg ml^−1^ bovine serum albumin, 10 mm creatine phosphate, 0.1 mg ml^−1^ creatine kinase, pH 7.5). Fluorescence intensity was read every 30 s for 1 h (excitation, 485 nm/5 nm bandwidth; emission, 535 nm/10 nm bandwidth). Protein amounts were confirmed by comparing the fluorescence intensity of the reaction in each well with calibration curves relating fluorescence intensity to protein concentration. Each assay was repeated at least three times. Initial degradation rates representing the slope of the decay curves at time 0 were calculated as the product of the amplitude, and the rate constant was determined by nonlinear fitting of the time-dependent fluorescence change to the equation describing single exponential decay to a constant offset using the software package KaleidaGraph (version 4.1, Synergy Software).

##### Yeast Expression

In *S. cerevisiae*, fluorescent proteins were expressed from a CEN plasmid (YCplac33) and His3 proteins from a 2-micron plasmid (pYES2), both with a URA3 selection marker. The plasmids were transformed into *S. cerevisiae* strain BY4741, which carries a deletion of the efflux pump Pdr5 (see supplemental Table S3 for genotype) (95) using Frozen-EZ Yeast Transformation II kit (Zymo Research).

In *S. pombe,* fluorescent proteins were expressed after integration into the genome using pDUAL-derived plasmids (96). 2 μg of plasmid were digested with NotI and purified (Qiagen). Frozen competent cells of *S. pombe* (see supplemental Table S3 for genotype) were thawed in a 30 °C water bath for 2 min, mixed with purified plasmid DNA, and 30% PEG 3350. The mixture was vortexed, incubated at 30 °C for 1 h, heat shocked at 43 °C for 15 min, and placed at room temperature for 10 min. The cells were then pelleted at 1600 × *g* for 3 min and resuspended in 500 μl of ½YE (0.25% yeast extract, 1.5% glucose). Cells were shaken for 1 h at 30 °C and spread onto synthetic medium lacking leucine. Clones with a single cassette integrated were validated by PCR.

The proteasome substrates and the RFP dsRed-Express2 (38) were expressed from separate promoters on the *S. cerevisiae* and *S. pombe* plasmids as specified. Proteasome activity was inhibited by 100 μm bortezomib. Protein synthesis was inhibited by 125 μm cycloheximide. 2% glucose was used to turn off protein expression from the promoter *gal1*.

##### Cell Culture Expression

Fluorescent proteins were expressed from the vector pCDNA5 (Life Technologies, Inc.) transiently transfected into HEK293 cells. The proteasome substrates and the RFP mCherry (97) were expressed from a single CMV promoter with the coding regions separated by an internal ribosome entry site derived from encephalomyocarditis virus (98).

HEK293 cells were cultured at 37 °C and 5% CO_2_ in DMEM supplemented with 10% FBS, 100 units/ml penicillin, and 100 units/ml streptomycin (Life Technologies, Inc.). Cells were seeded in a 6-well plate at 0.5 × 10^6^ cells/ml 24 h prior to transfection. 1 μg of DNA was transfected into cells with Lipofectamine 2000 (Life Technologies, Inc.) for 24 h. Cells were washed with PBS and recovered in complete DMEM for 24 h before analysis.

##### Yeast Growth Assay

His3 proteins were expressed from the inducible *gal1* promoter on a 2-micron plasmid (pYES2) with a URA3 selection marker in *S. cerevisiae* strain *pdr5*Δ (see supplemental Table S3 for genotype). Cells were grown with galactose as the carbon source at 30 °C to late log phase, serially diluted (*A*_600_ from 10^−1^ to 10^−6^) and stamped on plates with synthetic defined drop-out medium. Where indicated, 10 mm 3-AT was added to the medium as a competitive inhibitor of His3 to enhance assay sensitivity (55). Plates were incubated at 30 °C for 3 days for imaging.

##### Flow Cytometry

Yeast cells were grown at 30 °C to early log phase. HEK293 cells were grown for 24 h after transfection, trypsinized with TrypLE (Life Technologies, Inc.), washed, suspended in 0.5 ml of phosphate-buffered saline (PBS), and fixed by the addition of 0.5 ml of 3.7% formaldehyde in PBS. The fluorescence signals in the dsRed (or mCherry) and YFP (or GFP) channels were measured in a flow cytometer (LSR Fortessa, BD Biosciences) and analyzed by FlowJo software to calculate the medians of the YFP over RFP fluorescence ratios for each population. Each assay was repeated at least three times.

##### Western Blot

Yeast cells were grown to mid-log phase and lysed by vortexing with glass beads (BioSpec Products). Protein extracts were prepared and analyzed by Western blotting using standard protocols as described (28). YFP fusion proteins were detected with a mouse monoclonal anti-enhanced GFP antibody (1:1000, Clontech, catalog no. 632569) and an Alexa-800-labeled goat anti-mouse secondary antibody (1:20,000, Rockland Immunochemicals, catalog no. 610-132-121). Scs2 was detected by an anti-Scs2 rabbit polyclonal antibody (28) (1:1000, gift from J. Brickner, Northwestern University) and an Alexa-680 goat anti-rabbit secondary antibody (1:20,000, Invitrogen, catalog no. A21109). Protein amounts were estimated by direct infrared fluorescence imaging (Odyssey LI-COR Biosciences).

##### Bioinformatics and Statistical Analysis

The physicochemical properties of 115 sequences were calculated using the following tools: helix propensity, Agadir (99); backbone dynamics, DynaMine (63, 64); disorder propensity, IUPred (100); fraction of aliphatic, acidic, basic, nonpolar, aromatic or polar residues: EMBOSS pepstats (101); entropy, SEG (102, 103); flexibility, FLEXPLOT (62); and hydrophobicity, GRAVY (61). The charge density was calculated as the fraction of basic amino acids minus the fraction of acidic amino acids. Note that the flexibility prediction method of Vihenen *et al.* (62) was optimized for short windows of nine amino acid residues, although we calculated an aggregate flexibility score (mean flex (62)) by averaging the flexibility values for all consecutive nine-residue windows. To avoid making assumptions about the relationships between degradation rates and the numerical values of the sequence properties on their varied scales, we quantified the association of each metric with protein stability using the nonparametric Spearman rank correlation coefficients. Statistical significance of these associations was assessed by testing the hypothesis that a particular parameter does not correlate with degradation using pairwise *t* tests. The *p* values resulting from these tests were then used to compute false discovery rate *q*-values using the Benjamini-Hochberg method (60).

## Author Contributions

H. Y., A. K. S. G., S. R. W., D. W., G. K., K. M., M. W., S. C., and T. I. performed the experiments, analyzed the data, and co-wrote the paper. I. J. F., M. M. B., and A. M. directed the experiments, analyzed the data, and co-wrote the paper.

## Supplementary Material

Supplemental Data

## Footnotes

3 The abbreviations used are:

RFP: red fluorescent protein
DHFR: dihydrofolate reductase
3-AT: 3-amino-1,2,4-triazole
IQR: interquartile range.
