# Supplementary material for: Conserved Sequence Preferences Contribute to Substrate Recognition by the Proteasome
Source: J Biol Chem. 2016 May 17;291(28):14526–39. doi: 10.1074/jbc.M116.727578 (PMC4938175; doi:10.1074/jbc.M116.727578)
Supplement: Supplemental Data [file supp_291_28_14526__index.html]

Conserved Sequence Preferences Contribute to Substrate Recognition by the Proteasome — Conserved Sequence Preferences Contribute to Substrate Recognition by the Proteasome — Sequence Preferences in Proteasome Degradation — Supplemental Data 

# Conserved Sequence Preferences Contribute to Substrate Recognition by the Proteasome

## Supplemental Data

- Supplementary Tables (.docx, 103 KB) - Table S1 and S2: Amino acid sequences or proteasomal initiation sequences Table S3: Yeast strain genotypes
