# Supplementary material for: Conserved Sequence Preferences Contribute to Substrate Recognition by the Proteasome
Source: J Biol Chem. 2016 May 17;291(28):14526–39. doi: 10.1074/jbc.M116.727578 (PMC4938175; doi:10.1074/jbc.M116.727578)
Supplement: Supplemental Data [file 10.1074_M116.727578_jbc.M116.727578-1.docx]

Supplementary Tables for

**Conserved Sequence Preferences Contribute to Substrate Recognition by the Proteasome**

Houqing Yu^1,2^, Amit K. Singh Gautam^1^, Shameika R. Wilmington^1,2^, Dennis Wylie^3^, Kirby Martinez-Fonts^1,2^, Grace Kago^1^, Marie Warburton^1^, Sreenivas Chavali^4^, Tomonao Inobe^5^, Ilya J. Finkelstein^1^, M. Madan Babu^4^, and Andreas Matouschek^1,2^

^1^Department of Molecular Biosciences, The University of Texas at Austin, Austin, TX 78712

^2^Department of Molecular Biosciences, Northwestern University, Evanston, IL 60208

^3^Center for Computational Biology and Bioinformatics, The University of Texas at Austin, Austin, TX 78712

^4^Medical Research Council Laboratory of Molecular Biology, Cambridge, UK.

^5^Frontier Research Core for Life Sciences, University of Toyama, 3190 Gofuku, Toyama-shi, Toyama 930-8555, Japan

To whom correspondence should be addressed: Andreas Matouschek, Department of Molecular Biosciences, The University of Texas at Austin, 2506 Speedway Stop A5000, Austin, TX 78712, Tel.: (512) 232-4045; Fax: (512) 471-1218; Email: [matouschek@austin.utexas.edu](mailto:matouschek@austin.utexas.edu)

**Table S1.** Initiation region sequences.

| name | tail | amino acid sequences |
| --- | --- | --- |
| A | 35 | LRYQPLLRISQNCEAAILRASQTRLNTIHHHHHH |
| B | ODC | FPPEVEEQDDGTLPMSCAQESGMDRHPAACASARINV |
| C | polyG | GGGGGGGGGGGGGGGGGGGGGGGGGGGGGGGGGGGG |
| D | GRR | EIKDKEEVQRKRQKLMPNFSDSFGGGSGAGAGGGGMFGSGGGGGGTGSTGPGYSFPH |
| E | SRR | RSSSTSSDSGSSSSSSASSSSGSSSTSSDSGSSSSSSASSSSGSSSTSSDSGSSSSSSASSSSGSSSTSSDSGSSSSSSASSSSGSGTMKHGT |
| F | NB | RSKRIKCPDCEPFCNKRGSKRIKCPDCEPFCNKRGSKRIKCPDCEPFCNKRGS |
| G | NS | RSLIEEVRHRLKTTENSGSLIEEVRHRLKTTENSGSLIEEVRHRLKTTENSGS |
| H | SP1 | RSSLLTEVETPGSSLLTEVETPGSSLLTEVETPGSSLLTEVETPGSSLLTEVETP |
| I | SP2 | RSPESMREEYRKEGSPESMREEYRKEGSPESMREEYRKEGSPESMREEYRKEGSPESMREEYRKE |
| J | SPmix | RSPESMREEYRKEGSSLLTEVETPGSPESMREEYRKEGSSLLTEVETPGSPESMREEYRKE |
| K | SNS | RSPESMREEYRKEGSKRIKCPDCEPFCNKRGSPESMREEYRKE |
| L | NBS | RSKRIKCPDCEPFCNKRGSKRIKCPDCEPFCNKRGSPESMREEYRKE |
| M | DRR | RSDEDDDEDDDDDDDDEDDDDEGSDEDDDEDDDDDDDDEDDDDEGSGTMKHEYSIVSPK |
| N | eRR | GGGGAWLLPVSLVRRRTTLAPNTQTASPRALADS |
| O | PEST | GGSGGTLQMLPESEDEESYDTESEFTEFTEDELPYDDCVFGGQRLTL |
| P | Su9 | MASTRVLASRLASQMAASAKVARPAVRVAQVSKRTIQTGSPLQRAYSS |
| Q | no tail | N/A |

**Table S2.** Additional initiation region sequences analyzed in Figure 8.

| **No** | **SEQUENCE** | **YFP/RFP** |
| --- | --- | --- |
| 1 | RIPYRRYPRRRGPPRNYQQNYQNSESGEKNEGSESAPEGQAQQRRPYRRRRFPARVN | 0.27±0.09 |
| 2 | RIPKTVHGAEFYANKKHKGLPLNDANSRLQQNNSRHNLQEHNIDSSPCSEDSHARVN | 1.2±0.3 |
| 3 | RIPEKPYICKAPGCTKRYTDPSSLRKHVKTVHGAEFYANKKHKGLPLNDANSRARVN | 0.3±0.1 |
| 4 | RIPKNEGSESAPEGQAQQRRPYRRRRFPPYYMRRPYGRRPQYSNPPVQGEVMEARVN | 0.17±0.01 |
| 5 | RIPKSNAGMKHGTMDTESKKDPEGCDKSDDKNTVNLFGKVIETTEQDQEPSEAARVN | 5.5±0.8 |
| 6 | RIPTVGNGEVTLTYATGTKEESAGVQDNLFLEKAMQLAKRHANALFDYAVTGDARVN | 4.5±0.5 |
| 7 | RIPLGKMLGTSSPSIKSESDISSSNHHLVNGVRASDSLLTYSPDDLAENLNLDARVN | 0.6±0.1 |
| 8 | RIPDSTISTATTALALQARRNPAGTKWMEHVKLERLKQVNGMFPRLNPILPPKARVN | 0.14±0.01 |
| 9 | RIPAKRIVGSDSSPSNNNAGLHSQNSSLNSYTNMMRNINDEQLITEVIKSFKRARVN | 0.16±0.04 |
| 10 | RIPFRRGPPRQRQPREDGNEEDKENQGDETQGQQPPQRRYRRNFNYRRRRPENARVN | 0.6±0.2 |
| 11 | RIPDDKNTVNLFGKVIETTEQDQEPSEATVGNGEVTLTYATGTKEESAGVQDNARVN | 0.19±0.05 |
| 12 | RIPTGPGYSFPHYGFPTYGGITFHPGTTKSNAGMKHGTMDTESKKDPEGCDKSARVN | 2.6±0.9 |
| 13 | RIPPYYMRRPYGRRPQYSNPPVQGEVMEGADNQGAGEQGRPVRQNMYRGYRPRARVN | 0.21±0.02 |
| 14 | RIPQQDLSNTTSKREECLQVKTVKAEKPMTSQPSPGGQSSCSSQQSPISNYSNARVN | 1.0±0.3 |
| 15 | RIPPPPRDSGSHSQSRSPGRPTQGALGEQQDLSNTTSKREECLQVKTVKAEKPARVN | 0.7±0.2 |
| 16 | RIPKSTGPSPNQHSMNLNTSVLYSDEVLIQKVIKSLNINSNISICDSLGRTLLARVN | 0.16±0.02 |
| 17 | RIPSFPYGQPQYGVAGQYPYQLPKNNYNYYQTQNGQEQQSPNQGVAQHSEDSQARVN | 1.2±0.5 |
| 18 | RIPIQKVIKSLNINSNISICDSLGRTLLHLACLKNYSSLVYTLIKKGARVNDIARVN | 0.11±0.03 |
| 19 | RIPDSFGLTPLHFACISGDPKIIKMLLNCKVNYSLRSHNGLTAREVFIANHIHARVN | 0.13±0.01 |
| 20 | RIPHLACLKNYSSLVYTLIKKGARVNDIDSFGLTPLHFACISGDPKIIKMLLNARVN | 0.15±0.01 |
| 21 | RIPAEAANVTGPGGVPVQGSKYAADRNHYRRYPRRRGPPRNYQQNYQNSESGEARVN | 0.9±0.2 |
| 22 | RIPNGNASASTIGGSVLARQRFRGRLQTKGINSSTIMLCNIPESNRTFGISELARVN | 0.16±0.03 |
| 23 | RIPDGWNCDDDVDVADLPIVLRAMVNIGNGNASASTIGGSVLARQRFRGRLQTARVN | 0.19±0.02 |
| 24 | RIPNSYTNMMRNINDEQLITEVIKSFKRNNNLSTVNLSMCDVRGRTLLHLAAFARVN | 0.11±0.03 |
| 25 | RIPDARNIAKRIVGNDSPDSGTNGNSCSKSTGPSPNQHSMNLNTSVLYSDEVLARVN | 0.33±0.03 |
| 26 | RIPGADNQGAGEQGRPVRQNMYRGYRPRFRRGPPRQRQPREDGNEEDKENQGDARVN | 4.57±0.01 |
| 27 | RIPMTSQPSPGGQSSCSSQQSPISNYSNSGLELPLTDGGSIGDLSAIDETPIMARVN | 0.4±0.1 |
| 28 | RIPQSQQPQSQQSPQSQKQGNNVAAQQYYMYQNQFPGYSYPGMFDSQGYAYGQARVN | 0.21±0.07 |
| 29 | RIPNNENNNDDLPQEIEINDNKKAIFTYVDDTDRQLIELALQIVGLKMNGKLEARVN | 0.26±0.09 |
| 30 | RIPLQQNNSRHNLQEHNIDSSPCSEDSHLGKMLGTSSPSIKSESDISSSNHHLARVN | 0.5±0.2 |
| 31 | RIPQHQLQQQYLSQQQQYAQQQQQHPQPQSQQPQSQQSPQSQKQGNNVAAQQYARVN | 1.8±0.6 |
| 32 | RIPNGWQIISSSSGATPTSKEQSGSSTNGSNGSESSKNRTVSGGQYVVAAAPNARVN | 2.1±0.3 |
| 33 | RIPYMYQNQFPGYSYPGMFDSQGYAYGQQYQQLAQNNAQTSGNANQYNFQQGYARVN | 0.14±0.04 |
| 34 | RIPASTISSAYLSSRRSSGISPCFSSRRSSEASQAEGRPQNVSVADSYDPISTARVN | 0.33±0.01 |
| 35 | RIPAPAVSPLIGNGTQSNNTCSLGGPMTLLPGRSDLSGVDVTMLNMLNRRDSSARVN | 0.3±0.2 |
| 36 | RIPSGLELPLTDGGSIGDLSAIDETPIMDSTISTATTALALQARRNPAGTKWMARVN | 0.26±0.07 |
| 37 | RIPYYQTQNGQEQQSPNQGVAQHSEDSQQKQSQQQQQQQPQGQPQPEVQMQNGARVN | 4.28±0.01 |
| 38 | RIPVNGVRASDSLLTYSPDDLAENLNLDDGWNCDDDVDVADLPIVLRAMVNIGARVN | 0.22±0.06 |
| 39 | RIPNGGGAFSQARSSSTGSSSSTGGGGQESQPSPLALLAATCSRIESPNENSNARVN | 0.14±0.01 |
| 40 | RIPLPPVLVPRHSEYNPQHSLLAQFRNLGQNEPHMPLNATFPDSFQQPHSHPFARVN | 0.15±0.05 |
| 41 | RIPQYQQLAQNNAQTSGNANQYNFQQGYGQAGANTAAANLTSAAAAAAASPATARVN | 0.27±0.07 |
| 42 | RIPGQAGANTAAANLTSAAAAAAASPATAHAQPQQQQPYGGSFMPYYAHFYQQARVN | 0.28±0.09 |
| 43 | RIPGQNEPHMPLNATFPDSFQQPHSHPFPQSPNSSYPNSPGSSSSTYPHSPTSARVN | 0.7±0.2 |
| 44 | RIPNSQGPSQSGGTGELDLTATQLSQGANGWQIISSSSGATPTSKEQSGSSTNARVN | 4.89±0.01 |
| 45 | RIPPQSPNSSYPNSPGSSSSTYPHSPTSSDPGSPFQMPADTPPPAYLPPEDPMARVN | 0.9±0.7 |
| 46 | RIPSDPGSPFQMPADTPPPAYLPPEDPMAQDGSQPMDTNMTNMTAPTLPAEINARVN | 0.5±0.2 |
| 47 | NSGSHNFTTQQRKR | 3.1±0.2 |
| 48 | NSGSEDKKAGDEMKELREEIERLKLELSHKKDQETPNEDFKNELGG | 5.3±0.4 |
| 49 | NSGSQKVPSATNSKTTKSKANINNKSKKRGTNLVNKNSNSTPRQKKSQRYVSNLQRS | 2.7±0.2 |
| 50 | NSGSCSFENSNSTSIPSPASSSQSHTPMRNMSSLSDNSVFSRNMEQSSPITPSMGGETLMGG | 0.85±0.03 |
| 51 | NSNTEADSS | 4.9±0.4 |
| 52 | NSGSHKIRRMLSKELQDHRKVMNDVANKDVGEPSSEKLELNAEYTGKQFEHGG | 2.6±0.4 |
| 53 | NSGSTIQMRYGHSLPEARALNIEDCDLDRATNDFYLEPLIERDLLAHYDHQIVMDVRMVNLGG | 3±2 |
| 54 | NSGSKQLAHEEHINNDGDNDDENSNNIESSPLKQGHHHPKGQADDNNEGPDEEESTKEVPKPGGG | 2.3±0.9 |
| 55 | NSGSLRKRRQQQLSSNSTDNSLHPNSGQTPRASDSQRRHKKRNTTNLDRLRQEREENSLEMDCTQSGG | 1.0±0.6 |
| 56 | NSGSANVDSQSNNKHDGKDDDATNNNDGQDNNTNNDHNNNSNINNNNVGSHGISSHSPSSIRDTGG | 2.6±0.4 |
| 57 | NSGSLLSRRHGSATAKQRA | 2.7±0.4 |
| 58 | NSGSSQSINSANNIQSSDSDLVQHFESLAQEIRHHKKYKQNNSKQRKILKKIQDLKQTPPEATLGG | 0.26±0.02 |
| 59 | NSGSSPEQQVISEENDAKKLEQSALNSEASEDSEAMDEESKALKAAAEKADAPIDTNKMD | 1.9±0.9 |
| 60 | NSGSAKHQEISSAGTSSNTTKNVNNNKNDSNDDNNGNNNNDASNLMESVLDKTSSHSPSSIRDT | 2.0±0.2 |
| 61 | NSGSEQNKTT | 2.73±0.09 |
| 62 | NSGSEGIVTRLNETFNPEIQALPPLREIISGTSETHSSNNPFEIHSSNIDSELRNRFDYSEEEMDEDDDVDVFAGG | 2.8±0.1 |
| 63 | NSGSTSSLNEITPSKNRVTSACNSERRTTSQEANNLEGYHSCAQE | 3.0±0.2 |
| 64 | NSGSNTTSENIE | 3.0±0.1 |
| 65 | NSGSMKEAQKQLEEVQEQLTEYESQNLKLKKKLEATKTENSELQSTIVTLNTELENLKKNKKAQKKYS | 2.6±0.2 |
| 66 | NSGSLTVEIKEETSKD | 4.2±0.2 |
| 67 | NSGSCEGPSHGGLPGG | 3.2±0.1 |
| 68 | NSGSFRNPDSAFSANAKRGSKLVALRRINMEHIQQSRDNKQY | 0.82±0.05 |
| 69 | NSGSQQILSRQQSLGSAFGHSPPLIHPAPTFPTQRPIPGIPTVLGG | 0.17±0.02 |
| 70 | NSGSADQKTTGKDIGGAAVSSMSGCPVMHESSSSSPPSSECP | 2.8±0.1 |
| 71 | NSGSHGSLPHVAEPSVPYRGTVFAMDPRNGYMEPHYHPPHLFPAGG | 0.35±0.07 |
| 72 | NSGSRVTSMTQLNHHGRSPTSSPGNESSALLLTHSWVNKTSLHSVEADSS | 0.93±0.09 |
| 73 | NSGSNRFHRGKKSVERIRKFQNRQKNAKIKASDDAISKKSTSVNVSDGKIKRRDKKVSAGRTTVVVENTKGG | 1.4±0.1 |
| 74 | NSGSSHDDVANGIVPKHVVNVQNPPKQEVFEKIPSPEFNSNNEKELVQRKGSANEKLHQELGEKQPASGG | 4.2±0.2 |
| 75 | NSGSHLDLVKERPRFERSLEDLLKENATLAIELTKEITVSKRSSGEEKNDSETKGTHVERRRVPFLRDLLVKKRTTLKPRVPTLKRRRVPFLRDLLVKKRTTLKPRVPTLKRRRVPFLSLQSLPERLPEADSS | 0.22±0.02 |
| 76 | NSGSSWKDVQQEQEI | 4.2±0.2 |
| 77 | NSGSTDGTGDDHDGAPLSSSPSFGQQNDNSNWTCLKQGCF | 1.8±0.2 |
| 78 | NSGSERAKANLRAI | 1.9±0.3 |
| 79 | NSGSYHLQNDYSDAKTIVDTETEENLSTPLNLSTLNWKPQSWVLVCLTWVTTKPTNCLSTKLSTKRDLTLEPEELRLKKKTSVTL | 0.18±0.01 |
| 80 | NSGSTEDITVQEPVPLPEDAPEDAEPQFKEVTKTIKKDVLGMTAKTFALNPVELNDLIEKENELRNQDKLVAGG | 0.48±0.04 |
| 81 | NSGSTPWKVYHRNLERKVGPIEERS | 1.4±0.1 |
| 82 | NSGSMKRHVSSSFNNKVPLIKASSSNNSATSSPSIANSQLKSLENGG | 3.3±0.3 |
| 83 | NFNSHNVYITADKQKNGIKANFKIRHNVEDGGVQLADHYQQNTPIGDGPVLLPDNHYLSYQSKLSKDPNEKRDHMVLLEFVTAAGITHGMDELYRIRRLELINVT | 2.6±0.2 |
| 84 | NSGSLPPTMGKKTLLSLLPLLKTD | 0.5±0.1 |
| 85 | NSGSQYGLEAARSGGPSFGPGGPGGAGGAGGFPGGAGGFSGGHAFSNEDAFNIFSQFFGGSSPFGGPDDSVSLSLLTHLVVVLVWVVCQEADSS | 0.7±0.1 |
| 86 | NSGSPMEQIQKKQQELRQARQRMFRHER | 1.6±0.2 |
| 87 | NSGSMRCLAPRPAGFYLFEPQGFSQCATELGPLEGGYLELLNSDADPLCLYHFYDQMDLAGEEEIELYS EPDTDTINCDQFSRLLCDMEGDEETREAYANIAEGG | 1.0±0.1 |
| 88 | NSGSEFISLSPPHEALDYHFGLEEGEGIRDLFDCDFGDLTHWTSEADSS | 4.6±0.2 |
| 89 | NSGSELFQDLSQLQETWLAEAQVPDNDEQFVPDYQAESLVWEADSS | 2.0±0.1 |
| 90 | NSGSFVFTYPEADSFPPGGAGGAGVSQVVLVVSLVVTLSLTKTLSTSSLNSSVVLLHSVVLTTLVSLSLLTHLVVVLVWVVCQEADSS | 0.91±0.08 |
| 91 | NSGSMEVAPEQPRWMAHPAVLNAQHPDSHHPGLAHNYMEPAQLLPPDEVDVFFNHLDSQGNPYYANPAHARARVGG | 0.19±0.02 |
| 92 | NSGSSSPSMRPDVSSPPSSSSTATTGPPPKLCLVCSDEASGCHYGG | 1.5±0.2 |
| 93 | NSGSEFKLELVEKLFAEDTEAKNPFSTQDTDLDLEMLAPYIPMDDDFQLRSFDQLSPLESSSASPESASPQSTVGG | 1.0±0.1 |
| 94 | NSGSDIGLSLQRVFTDLKNMDATWLDSLLTPVRLPSIQAIPCAPGG | 0.14±0.01 |
| 95 | NSGSLPQQATYFPPSPPSSEPGSPDRQAEMLQNLTPPPSYAATIASKLAIHNPNLPTTLPGG | 0.34±0.04 |
| 96 | NSGSMDFFRVVENQQPPATMPLNVSFTNRNYDLDYDSVQPYFYCDEEENFYQQQQQSELQPPAPSEDIWKKFELLPTPPLSPSRRSGLCSPSYVAVTPFSLRGDNDGGGGSFSTADQLEMVTELLGGDMVNQGG | 0.37±0.06 |
| 97 | NSGSIESLQELLREQVENYYSLPGQSCSEPTSPTSNCSDGMPECGG | 3.4±0.4 |
| 98 | NSGSMEEPQSDPSVEPPLSQETFSDLWKLLPENNVLSPLPSQAMDDLMLGG | 0.81±0.05 |
| 99 | NSGSDVSKLGEEEDEISPLSHDNFQYESEENGNPSPPIYKKSGELVKSSLKRRSKSLPITPKSIFNKTGSKGG | 0.73±0.02 |

**Table S3.** Yeast Strains.

| **strain** | **genotype** | **source** |
| --- | --- | --- |
| BY4741 | *MAT***a** *his3Δ1 leu2Δ0 met15Δ0 ura3Δ0* | (1) |
| *pdr5∆* | BY4741 with *pdr5::kanMX4* | (2) |
| MAHQ1 | *pdr5∆* with *uba1-204* | this study |
| YYS40 | *MAT***a** *RPN11-3* × *FLAG-HIS3 ade2 can1 his3 leu2 trp1 ura3 ssd1* | (3) |
| *S. pombe* | *h+ his+ ade6-M216 leu1-32 ura4-D18 arg+* | (4) |

References

1. Brachmann, C. B., Davies, A., Cost, G. J., Caputo, E., Li, J., Hieter, P., and Boeke, J. D. (1998) Designer deletion strains derived from Saccharomyces cerevisiae S288C: a useful set of strains and plasmids for PCR-mediated gene disruption and other applications. *Yeast* **14**, 115–132

2. Fleming, J. A., Lightcap, E. S., Sadis, S., Thoroddsen, V., Bulawa, C. E., and Blackman, R. K. (2002) Complementary whole-genome technologies reveal the cellular response to proteasome inhibition by PS-341. *Proc. Natl. Acad. Sci. USA* **99**, 1461–1466

3. Saeki, Y., Isono, E., and Toh-E, A. (2005) Preparation of ubiquitinated substrates by the PY motif-insertion method for monitoring 26S proteasome activity. *Meth. Enzymol*. **399**, 215–227

4. Leupold, U. (1950) Die Vererbung von Homothallie und Heterothallie bei *Schizosaccharomyces pombe*. *CR Trav. Lab. Carlsberg Ser. Physiol*. **24**, 381–480
